# Three-dimensional electroanatomic mapping characteristics of superior vena cava myocardial sleeve and sinoatrial node in patients with atrial fibrillation

**DOI:** 10.3389/fcvm.2022.902828

**Published:** 2022-09-07

**Authors:** Xinguang Chen, Yao Lu, Yanmin Liu, Qiushi Chen, Hongwu Chen, Weizhu Ju, Gang Yang, Yeqian Zhu, Pengcheng Zhao, Jian Zhang, Yangming Mao, Xiaoling Su, Fengxiang Zhang, Minglong Chen

**Affiliations:** ^1^Section of Pacing and Electrophysiology, Division of Cardiology, The First Affiliated Hospital With Nanjing Medical University, Nanjing, China; ^2^Section of Pacing and Electrophysiology, Division of Cardiology, The First Affiliated Hospital of Gannan Medical University, Ganzhou, China; ^3^Department of Cardiology, Qinghai Province Hospital, Xining, China

**Keywords:** atrial fibrillation, sinoatrial node, superior vena cava, myocardial sleeve, the earliest activation

## Abstract

**Background:**

Three-dimensional activation mapping during sinus rhythm can demonstrate the earliest atrial activation (EAA) site, which could be the sinoatrial node (SAN). We aimed to compare the electroanatomical characteristics of superior vena cava (SVC), myocardial sleeve, and SAN between patients with atrial fibrillation (AF) and non-AF.

**Materials and methods:**

In this study, 136 patients with AF were assigned to the study group, and 20 patients with premature ventricular contractions (PVCs) who had no history of AF were assigned to the control group. The right atrium (RA) and SVC anatomical activation models were constructed, and the EAA of SAN was delineated using the CARTO3 mapping system. The length of the SVC myocardial sleeve (LSVC) was measured.

**Results:**

Of the 136 patients, 93 patients had paroxysmal AF (PAF), and 43 patients had persistent AF (PsAF). The LSVC was not significantly different among AF and non-AF, PAF, and PsAF. The LSVC in men was longer than in women (42.1 ± 9.4 mm vs. 35.4 ± 8.1 mm, *p* < 0.001). The LSVC was longer in patients with EAA of SAN above the RA-SVC junction than in those with below the RA-SVC junction (*p* < 0.001). The EAA of SAN was below the RA-SVC junction in 64/136 (47.1%) and was above the junction in 72/136 (52.9%) patients with AF. The spatial distribution of the EAA of SAN between PAF and PsAF was not different. There was a trend of statistical difference in the distribution of the EAA of SAN between PsAF and non-AF.

**Conclusion:**

The EAA of SAN was located in the SVC in most of the patients, especially in patients with PsAF.

## Introduction

Pulmonary veins (PVs) isolation is the cornerstone of radiofrequency ablation of atrial fibrillation (AF) because ectopic foci arising from PVs are the primary sources for the initiation and maintenance of AF (1). Nevertheless, ectopic activities from non-PV foci can also contribute to the occurrence of AF (2, 3). The superior vena cava (SVC) appears to be the most common source of ectopics of AF, accounting for 26–30% of non-PV foci (4). It has been reported that the adjunctive role of superior vena cava isolation (SVCI), in addition to pulmonary veins isolation (PVI), can improve the outcome of AF ablation (5–7). Recently, the standard electrical SVCI method is ablating 5–10 mm above the SVC-right atrium (RA) junction defined fluoroscopically using multiple projections of the SVC angiography. But unfortunately, the SVCI may result in potential sinoatrial node (SAN) injury, with a risk rate of 2–4.5%, especially when the procedure is performed under an inaccurate delineation of the SVC-RA junction near the SAN location (8, 9).

Additionally, the SAN location shows great heterogeneity, 10–25% of patients with AF showed the SAN above the SVC-RA junction (10), which undoubtedly increases the risk of SAN injury. To safely and effectively perform the SVCI without SAN dysfunction, identifying the SAN location at the level of the SVC-RA junction and the location of the SAN is essential. The activation mapping utilizing a 3D mapping system during sinus rhythm could demonstrate the earliest activation (EAA) that could be localized as the SAN (11). This method has been successfully applied to modify SAN in patients with inappropriate SAN tachycardia but is limited to a few AF (8, 12). This study aimed to (1) compare the electroanatomical characteristics of SVC, myocardial sleeve, and SAN between patients with and without AF by using a three-dimensional electroanatomical mapping system (Carto3); and (2) provide the anatomical basis for the design of the SVC isolation pathway and improve the safety of SVCI.

## Materials and methods

### Patient population

In this study, one hundred thirty-six patients with AF who underwent radiofrequency catheter ablation for the first time at the first affiliated hospital of Nanjing medical university were assigned to the study group. Furthermore, 20 patients with premature ventricular contractions (PVCs) who had no history of AF and excluded structural heart disease were regarded as the control group. The exclusion criteria were left atrial diameter >55 mm, left ventricular ejection fraction (LVEF) <35%, New York Heart Association functional classes III or IV, contraindications for anticoagulation, prior AF ablation, left atrial thrombus, or presence of SVC stenosis, and previous SAN dysfunction. The study procedure complied with the principles of the Declaration of Helsinki and was approved by the Ethics Committee of the First Affiliated Hospital of Nanjing Medical University. Written informed consent was obtained from all the participants.

### Electrophysiology study

All antiarrhythmic drugs except amiodarone were discontinued more than five half-lives before the ablation procedure. The entire procedure was performed in a conscious or deep, sedated state. All patients with AF received anticoagulation therapy for at least 1 month. Before the procedure, transesophageal echocardiography or contrasted computed tomography was performed to exclude intracardiac thrombus. A 6F decapolar catheter (St. Jude Medical, Inc., St. Paul, MN, United States) was positioned *via* the left femoral vein in the coronary sinus. A 6F quadripolar catheter (St. Jude Medical, Inc.) was advanced into the right ventricle *via* a femoral vein. In addition, two 8.5 F long sheaths (SL1, St. Jude Medical, Inc) were advanced to the left atrium (LA) through a standard transseptal puncture. A deflectable decapolar circular catheter (Biosense Webster, United States.) was advanced through the sheath for PV mapping, and a deflectable quadripolar open irrigated SmartTouch (ST) catheter (Biosense Webster, United States) was inserted into the LA for mapping and ablation. After a transseptal puncture, intravenous heparin was administered to maintain an activated clotting time of 300–350 s. Activated clotting time was monitored every 60 min, and the heparin dose was adjusted accordingly. Intracardiac electrograms were recorded using a digital electrophysiological recording system (Bard CardioLab, Inc., United States) and were filtered from 30 to 300 Hz. The three-dimensional anatomical or activation mapping mode of RA-SVC reconstruction was guided by the CARTO3 three-dimensional mapping system (BioSense Webster, United States) under the premise of sinus rhythm. Direct current cardioversion was performed if patients with AF did not restore sinus rhythm after bilateral circumferential pulmonary vein isolation (CPVI). The RA-SVC model construction and activation mapping were performed in the control group after the corresponding type of procedure.

### Right atrium-superior vena cava mapping

As for patients with AF, electroanatomical geometry of the RA and SVC was constructed using the ST ablation catheter with the CARTO3 three-dimensional mapping system (BioSense Webster, United States) after completing CPVI. In the control group, the RA-SVC model construction and activation mapping were performed after the corresponding type of procedure. The ST catheter can quantitatively display the attachment strength between the catheter tip and tissue in real-time. During the modeling process of the ablation catheter, the contact force was kept at 3–5 g to avoid a false cavity, and the complete anatomical models of RA and SVC were constructed. The RA-SVC junction was defined as the ostium of the SVC where the diameter of the SVC abruptly changed on the created geometry from the cranial to caudal direction.

### Superior vena cava mapping

**FIGURE 1:**
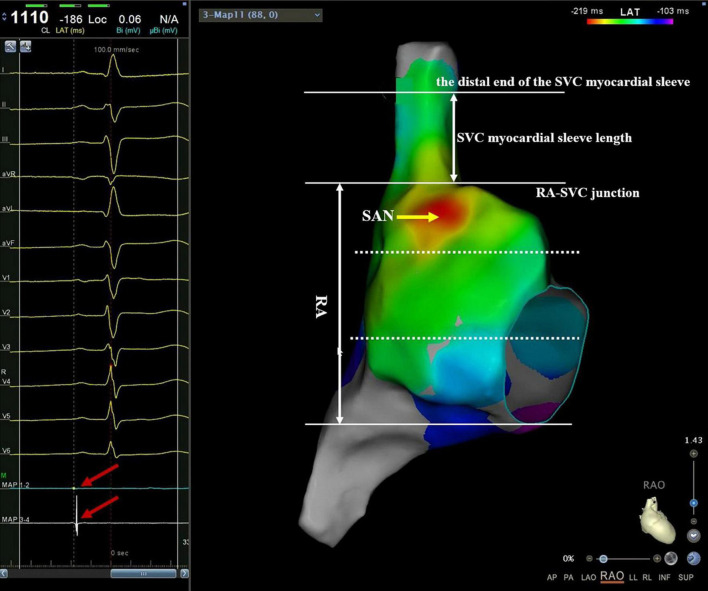
An example of mapping catheter-guided SVC activation mapping. Manipulating the mapping catheter along with the RA from the caudal to the cranial direction to reconstruct the 3D activation map of RA and SVC. At the junction of RA-SVC, both the proximal 1–2 electrode and the distal 3–4 electrode could record the SVC potential. Then, the catheter was slowly moved toward the cranial direction with 3–5 g pressure. When the SVC potential of the proximal electrode of the catheter just disappeared, and at the same time, the SVC potential of the distal electrode still existed (red arrow on the left plan), the plane of the proximal electrode of the catheter was indicated the end of SVC musculature sleeve. The myocardial sleeve length of SVC was defined as the vertical distance from the junction of RA-SVC to the distal end of the myocardial sleeve. The RA was divided into three equal parts in the right anterior oblique position, namely, high, middle, and low RA. Note that the red region indicates the location of the SAN. RA, right atrium; SVC, superior vena cava.

The activation mapping was performed during stable sinus rhythm to localize the actual site of the SAN. If AF was still sustainable during mapping, sinus rhythm was restored with cardioversion. The sequential recording was then performed at multiple points by manipulating the catheter along the SVC circumference to reconstruct the 3D activation map of RA and SVC. A bipolar electrogram from the coronary sinus was used as the timing reference, and the local activation time was annotated manually at the onset of the bipolar spike. For patients with sinus rhythm, the RA was predominantly activated by the discharges from SAN. Therefore, the SAN region was tagged as the earliest breakthrough of RA on the activation map (13). To reveal the location of SAN, we divided it into two regions: above the RA-SVC junction (namely, within SVC) and below the RA-SVC junction, with the RA-SVC junction as the boundary. For the former, we divided the SVC into four aspects: anterior, lateral, posterior, and septal. For the latter, the RA was divided into three equal parts in the right anterior oblique position, namely, high, middle, and low RA. Meanwhile, the vertical distance from the EAA point of SAN to the junction of RA-SVC was measured. If the EAA of SAN was located above the RA-SVC junction, the vertical distance between them was recorded as a positive value, and if it was located at the RA-SVC junction or below, the vertical distance between them was recorded as zero or a negative value, respectively (Figure 1).

### Mapping and measurement of superior vena cava myocardial sleeve

After constructing RA and SVC anatomical models, the catheter was gradually manipulated from the caudal to the cranial direction along the SVC circumference. At the junction of RA-SVC, both the proximal 1–2 electrode and the distal 3–4 electrode could record the SVC potential, and then the catheter was slowly moved toward the head with 3–5 g pressure. When the SVC potential of the proximal electrode of the catheter just disappeared and that of the distal electrode still existed, the plane of the proximal electrode of the catheter was defined as the distal end of the SVC myocardial sleeve and tagged in the 3D anatomical model. The length of the SVC myocardial sleeve (LSVC) was defined as the vertical distance from the junction of RA-SVC to the distal end of the myocardial sleeve in the 3D mapping system (Figure 1).

### Statistical analysis

All data were expressed as mean ± standard deviation (SD). Continuous variables were compared using Student’s *t*-test. Categorical variables were presented as percentages and compared by χ^2^ tests or Fisher’s exact tests. All statistical tests and confidence intervals were two-sided, with a significance level of 0.05.

## Results

### Basal characteristics

A total of 136 patients with AF (PAF and PsAF) from January 2019 to January 2021 were included in this study. Basal characteristics between the PAF and PsAF were balanced except for age, left axis deviation (LAD), right axis deviation (RAD), and LVEF. The mean age was 58.7 ± 8.1 years, 48 patients (35.3%) were women, and 43 (31.6%) patients were PsAF in Table 1. Furthermore, twenty patients with non-AF were assigned to the control group. Among them, 5 were men, and 15 were women, with an average age of 50.3 ± 13.5 years. All of them had no obvious structural heart disease. General information is listed in Table 2.

**TABLE 1:** Baseline characteristics of patients with PAF and PsAF.

Variables	PAF (*n* = 93)	PsAF (*n* = 43)	*P*-value
Age (years)	57.7 ± 7.9	61.0 ± 8.4	0.03
Male, *n* (%)	60 (64.5)	28 (65.1)	0.95
Height (cm)	167.6 ± 7.5	167.5 ± 7.5	0.99
Weight (kg)	69.7 ± 9.6	71.3 ± 12.3	0.42
BMI (kg/m^2^)	24.8 ± 2.6	25.3 ± 3.8	0.37
Smoke, *n* (%)	25 (26.9)	14 (32.6)	0.50
Alcohol, *n* (%)	25 (26.9)	9 (20.9)	0.46
Hypertension, *n* (%)	52 (55.9)	23 (53.5)	0.80
Diabetes, *n* (%)	11 (11.8)	5 (11.6)	0.97
CAD, *n* (%)	8 (8.6)	7 (16.3)	0.30
Stroke, *n* (%)	4 (4.3)	5 (11.6)	0.22
LAD (mm)	36.8 ± 3.8	42.0 ± 4.5	<0.001
RAD (mm)	33.1 ± 3.3	40.0 ± 4.6	<0.001
LVDD (mm)	47.2 ± 2.9	45.6 ± 5.3	0.34
LVDS (mm)	31.1 ± 2.4	31.8 ± 4.4	0.34
LVEF (%)	63.2 ± 3.3	60.6 ± 4.1	0.04
Amiodarone, *n* (%)	85 (91.4)	36 (83.7)	0.30
Beta-blocker, *n* (%)	80 (86.0)	33 (76.7)	0.18
Propafenone, *n* (%)	10 (10.8)	7 (16.3)	0.37
ACEI/ARB, *n* (%)	48 (51.6)	20 (46.5)	0.23
CCB, *n* (%)	4 (4.3)	3 (7.0)	0.68
Statin, *n* (%)	20 (21.5)	11 (25.6)	0.60
Hypoglycemic drugs, *n* (%)	6 (6.5)	5 (11.6)	0.49
NOAC, *n* (%)	90 (96.8)	40 (93.0)	0.38

BMI, body mass index; CAD, coronary heart disease; LAD, left atrial diameter; RAD, right atrial diameter; LVDD, left ventricular end-diastolic diameter; LVDS, left ventricular end systolic diameter; LVEF, left ventricular ejection fraction; NOAC, new oral anticoagulants.

**TABLE 2:** Baseline characteristics of patients with AF and non-AF.

Variables	AF (*n* = 136)	Non-AF (*n* = 20)	*P*-value
Age (years)	58.7 ± 8.1	50.3 ± 13.5	<0.001
Male, *n* (%)	88 (64.7)	5 (25)	0.002
Height (cm)	167.6 ± 7.5	162.8 ± 4.5	0.01
Weight (kg)	70.2 ± 10.5	60.1 ± 5.6	<0.001
BMI (kg/m^2^)	24.9 ± 3.0	22.7 ± 2.5	0.002
Smoke, *n* (%)	39 (28.7)	2 (10.0)	0.13
Alcohol, *n* (%)	34 (25.0)	2 (10.0)	0.23
Hypertension, *n* (%)	75 (55.1)	8 (40)	0.31
Diabetes, *n* (%)	16 (11.8)	1 (5.0)	0.60
CAD, *n* (%)	15 (11.0)	0 (0)	0.25
Stroke, *n* (%)	9 (6.6)	0 (0)	0.50
LAD (mm)	38.4 ± 4.7	34.5 ± 4.0	<0.001
RAD (mm)	35.4 ± 4.9	30.0 ± 2.5	<0.001
LVDD (mm)	47.2 ± 4.2	45.8 ± 6.4	0.20
LVDS (mm)	31.6 ± 3.3	31.6 ± 4.6	1.00
LVEF (%)	62.7 ± 3.8	62.5 ± 5.5	0.84

BMI, body mass index; CAD, coronary heart disease; LAD, left atrial diameter; RAD, right atrial diameter; LVDD, left ventricular end-diastolic diameter; LVDS, left ventricular end systolic diameter; LVEF, left ventricular ejection fraction.

### The length of the SVC myocardial sleeve in patients with atrial fibrillation and non-atrial fibrillation

**FIGURE 2:**
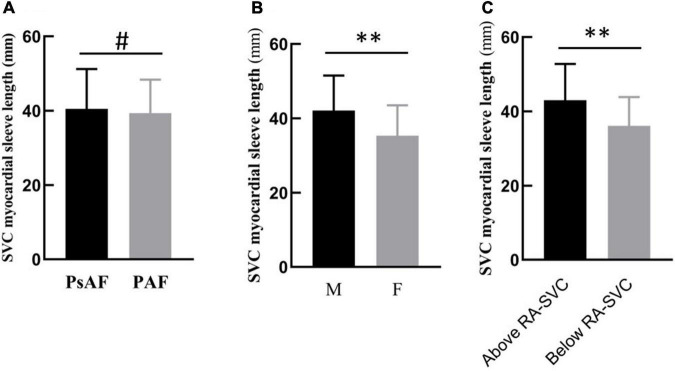
Subgroup analysis of LSVC in patients with atrial fibrillation (AF). **(A)** There was no difference between PAF and PsAF. **(B)** The length of the myocardial sleeve in men was longer than in women. **(C)** When the location of SAN was above the RA-SVC junction, the length of the SVC myocardial sleeve was longer than that below the junction. PAF, paroxysmal atrial fibrillation; PsAF, persistent atrial fibrillation; M, male; F, female; RA, right atrium; SAN, sinus node; SVC, superior vena cava, ^#^*p* > 0.05; **p < 0.01.

The LSVC was not significantly different between patients with AF and non-AF (39.8 ± 9.5 mm vs. 35.7 ± 8.5 mm, *p* = 0.07). There was no difference in the LSVC between patients with PAF and PsAF (39.4 ± 9.0 mm vs. 40.5 ± 10.7 mm, *p* = 0.51). However, in patients with AF, the LSVC was significantly longer in men than in women (42.1 ± 9.4 mm vs. 35.4 ± 8.1 mm, *p* < 0.001). The LSVC was longer in patients with EAA of SAN above the RA-SVC junction than in those with below the RA-SVC junction (*p* < 0.001) (Figure 2).

### Factors associated with length of the SVC myocardial sleeve

**FIGURE 3:**
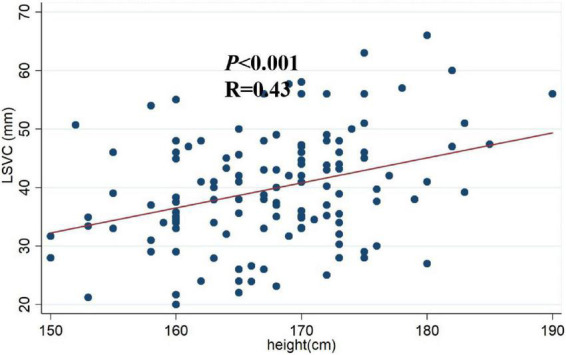
Scatter plot of height and LSVC. A significant linear relationship between myocardial sleeve length and height (*R* = 0.43, *p* < 0.001). LSVC: SVC myocardial sleeve length.

The Pearson correlation analysis shows that the LSVC was positively correlated with height (*r* = 0.34, *p* < 0.01) (Table 3); However, there was no significant correlation between the LSVC and the cardiac parameters, such as RAD, but a significant linear relationship between LSVC and height (*R* = 0.43, *p* < 0.001) (Figure 3). After adjusting for gender and weight, height was still a risk factor for the LSVC (Table 4).

**TABLE 3:** Correlation analysis of LSVC.

	RAD	LAD	RVDd	LVDD	LVDS	Height
**LSVC**	***p* = 0.54**	***p* = 0.97**	***p* = 0.64**	***p* = 0.34**	***p* = 0.42**	***p* < 0.001**
	*r* = 0.05	*r* = −0.003	*r* = 0.04	*r* = 0.08	*r* = 0.07	*r* = 0.34

LSVC, SVC myocardial sleeve length; LAD, left atrial diameter; RAD, right atrial diameter; RVDd, right ventricular end-diastolic diameter; LVDD, left ventricular end-diastolic diameter; LVDS, left ventricular end systolic diameter; LVEF, left ventricular ejection fraction.

**TABLE 4:** Multiple linear regression analysis of LSVC.

Variables	Standard error	Standardized coefficient	*t*	*p*
Sex	2.12	0.20	1.90	0.06
Weight	0.09	−0.14	−1.33	0.19
Height	0.16	0.29	2.28	0.02

### Distribution of earliest atrial activation of sinoatrial node in patients with atrial fibrillation

**FIGURE 4:**
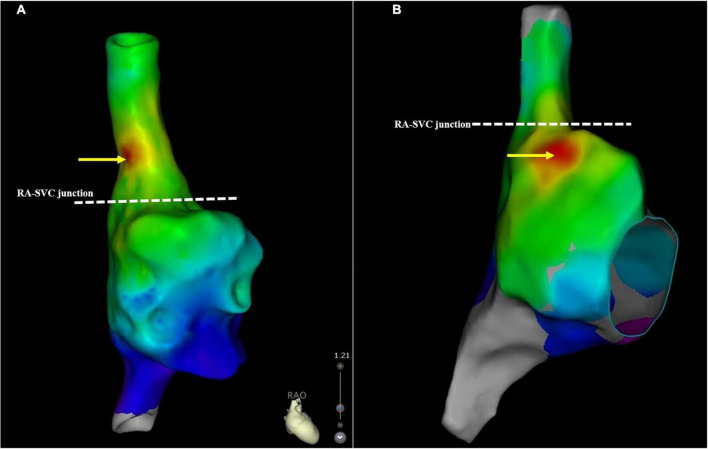
A schematic illustration demonstrating the SAN location. **(A)** This case demonstrates the SAN located above the RA-SVC junction. **(B)** The SAN was below the RA-SVC junction. The yellow arrow indicates the location of the SAN. The white dotted line indicates the RA-SVC junction. RA, right atrium; SAN, sinus node; SVC, superior vena cava.

The EAA of SAN was located below the RA-SVC junction in 64 (47.1%) patients with AF, of which it was located in high RA in 60 (93.8%) patients with AF and in the middle RA of the remaining 4 (6.2%) patients with AF. The average distance between the EAA of SAN and RA-SVC junction was −8.9 ± 6.1 mm. The EAA of SAN was located above the RA-SVC junction in 72/136 (52.9%) patients with AF, of which it was located in the lateral wall in 46 (63.9%), anterior wall in 14 (19.4%), septal aspect in 8 (11.1%), and posterior wall in 4 (5.6%) patients. The mean distance between the EAA of SAN and the RA-SVC junction was 10.2 ± 4.7 mm. There was no significant difference in the spatial distribution of the EAA and the distance between EAA and RA-SVC junction for patients with PAF and PsAF. The SAN was predominantly located in the high RA in patients with EAA of SAN below the RA-SVC junction. In patients with EAA of SAN located in the SVC, the SAN was mainly found on the lateral wall/aspect (Figure 4 and Table 5).

**TABLE 5:** Distribution of EAA of SAN in patients with AF.

	All patients (*n* = 136)	PAF (*n* = 93)	PsAF (*n* = 43)	*P*
SAN location distribution				0.23
SAN above RA-SVC junction, *n* (%)	72 (52.9)	46 (49.5)	26 (60.5)	
SAN below RA-SVC junction, *n* (%)	64 (47.1)	47 (50.5)	17 (39.5)	
SAN above RA-SVC junction, *n* (%)	72 (52.9)	46 (49.5)	26 (60.5)	0.82
Lateral, *n* (%)	46 (63.9)	30 (65.2)	16 (61.5)	
Anterior, *n* (%)	14 (19.4)	9 (19.6)	5 (19.2)	
Septal, *n* (%)	8 (11.1)	4 (8.7)	4 (15.4)	
Posterior, *n* (%)	4 (5.6)	3 (6.5)	1 (3.9)	
Distance of RA-SVC junction-SAN, mm	10.2 ± 4.7	9.7 ± 4.7	11.2 ± 4.8	0.20
SAN below RA-SVC junction, *n* (%)	64 (47.1)	47 (50.5)	17 (39.5)	0.57
High RA, *n* (%)	60 (93.8)	43 (91.5)	17 (100)	
Middle RA, *n* (%)	4 (6.2)	4 (8.5)	0 (0)	
Distance of RA-SVC junction-SAN (mm)	−8.9 ± 6.1	−9.1 ± 6.3	−8.6 ± 5.6	0.78

SVC, superior vena cava; RA, right atrium; SAN, sinoatrial node.

### Distribution of earliest atrial activation of sinoatrial node in patients with non-atrial fibrillation

The EAA of SAN was below the RA-SVC junction in 13/20 (65.0%) patients with non-AF, of which it was located in high RA in 11 (84.6%) and in the median RA of the remaining 2 (15.4%) patients with non-AF. The average distance between the EAA and the RA-SVC junction was −9.0 ± 4.9 mm. In 7/20 (35%) patients with non-AF, the EAA was located above the RA-SVC junction. In 4 of them (57.1%), it was located in the lateral wall of the SVC. In 2 (28.6%) patients, it was located in the anterior wall and in the posterior wall of the remaining 1 (14.3%) patient. The mean distance between the EAA and the RA-SVC junction was 9.5 ± 6.2 mm (Table 6).

**TABLE 6:** Comparison of the distribution of EAA of SAN between AF and non-AF patients.

	AF (*n* = 136)	Non-AF (*n* = 20)	*P*
SAN location distribution			0.13
SAN above RA-SVC junction, *n* (%)	72 (52.9)	7 (35.0)	
SAN below RA-SVC junction, *n* (%)	64 (47.1)	13 (65.0)	
SAN above RA-SVC junction, *n* (%)	72 (52.9)	7 (35.0)	0.72
Lateral, *n* (%)	46 (63.9)	4 (57.1)	
Anterior, *n* (%)	14 (19.4)	2 (28.6)	
Septal, *n* (%)	8 (11.1)	0 (0)	
Posterior, *n* (%)	4 (5.6)	1 (14.3)	
Distance of RA-SVC junction-SAN, mm	10.2 ± 4.7	9.5 ± 6.2	0.69
SAN below RA-SVC junction, *n* (%)	64 (47.1)	13 (65.0)	0.27
High RA, *n* (%)	60 (93.8)	11 (84.6)	
Middle RA, *n* (%)	4 (6.2)	2 (15.4)	
Distance of RA-SVC junction-SAN (mm)	−8.9 ± 6.1	−9.0 ± 4.9	0.99

SVC, superior vena cava; RA, right atrium; SAN, sinoatrial node.

### Comparison of the distribution of earliest atrial activation of sinoatrial node between patients with atrial fibrillation and non-atrial fibrillation

The distribution of the EAA of SAN among patients with AF and non-AF, and patients with PAF and non-AF showed no difference, respectively. However, there was a trend of statistical difference in the distribution of the EAA of SAN and the relative distance between the EAA and the RA-SVC junction between patients with PsAF and non-AF (Tables 6, 7).

**TABLE 7:** Comparison of the distribution of EAA of SAN between PsAF and non-AF patients.

	PsAF (*n* = 43)	Non-AF (*n* = 20)	*P*
SAN location distribution			0.06
SAN above RA-SVC junction, *n* (%)	26 (60.5)	7 (35.0)	
SAN below RA-SVC junction, *n* (%)	17 (39.5)	13 (65.0)	
Relative distance of RA-SVC junction-SAN (mm)	3.4 ± 11.0	−2.5 ± 10.4	0.05

SVC, superior vena cava; RA, right atrium; SAN, sinoatrial node.

## Discussion

### Major findings

The major findings in the current study are: (1) the LSVC in patients with AF and non-AF patients, PAF and PsAF were not different. (2) Height was a risk factor for the LSVC. (3) The EAA of SAN was located below the RA-SVC junction in some patients, but surprisingly, in most of the patients, it was located above the RA-SVC junction; that is, within the SVC, especially for patients with PsAF, the proportion was as high as 60.5%. (4) The SAN was predominantly located in the high RA in patients with EAA of SAN below the RA-SVC junction. In patients with EAA of SAN within the SVC, it was mostly located in the anterior and lateral wall of the SVC.

Previous studies have demonstrated that empirical SVCI combined with PVI could improve the outcome of PAF (5–7). During the SVCI, major concerns are the unintentional ablation of the SAN and the phrenic nerve. To avoid phrenic nerve palsy, a pacing maneuver was conventionally employed for localizing the phrenic nerve and proved simple and reliable (14). While the SAN site was mainly localized *via* the fluoroscopy delineation, this largely depends on the operator’s experience and lacks accuracy. Traditional circular mapping catheter-guided isolation is useful for SVCI (15). However, if the circular mapping catheter is placed diagonally against the SVC, the EAA site may be close to the SAN. For safe and effective SVCI, it requires knowledge of the locations of the SAN. Within this context, after delineation of SAN anatomical sites defined as the EAA revealed by the activation mapping, the electrical connection around the RA-SVC junction was ablated under the guidance of a mapping catheter.

### Myocardial extension into the superior vena cava

The mean length of the myocardial sleeve inside the SVC in the present study measured by electroanatomic mapping was 39.8 ± 9.5 mm. In addition, Spach et al. observed that excitation extended 20–50 mm above the junction of SVC with the base of the right atrial appendage in patients undergoing cardiac surgery (16). A human histological study demonstrated that cardiac myocytes in the RA wall extend to the SVC venous walls and form a muscular layer, covering a length of 45 mm (17). The histological finding of this study was comparable to the electrically activated sleeve length in our study. Conversely, Kholová et al. reported that myocardial sleeves were recognized in 19 out of 25 SVCs (76%), with a mean length of 13.7 mm (maximum, up to 47 mm) (18). The measurements are discordant with findings in our study on account of the heterogeneity of the study population. All patients in our study had a definite history of AF, while the proportion in Kholová’s study was only 28%.

Moreover, Higuchi et al. demonstrated that the SVC sleeve was longer in patients with arrhythmogenic SVC than in those without (34.7 ± 4.4 mm vs. 16.6 ± 11.4 mm, *p* < 0.0001) (19). Similarly, Tsai et al. performed electrophysiologic mapping of the SVC in patients with AF, and the mean distance determined by the electrical measurement was 33 ± 7 mm, ranging from 24 to 44 mm (20). More recently, ultra-high-resolution human SVC mapping conducted by Miyazaki et al. demonstrate that SVC musculature sleeve length was asymmetric and longest at the anteroseptal SVC (27.0–28.0 mm) and was shortest at the posterolateral SVC (22.0–23.0 mm) (21). This disparity between the mapping and morphological data may be explained by the bias attributable to the selection of patients with AF, variation in defining the RA-SVC junction, and potential for recording local electro-potential extending beyond the gross anatomic visible myocardial sleeve.

### The location of the sinoatrial node

Histologically, the shape of the SAN is more reminiscent of a tadpole with a head, body, and tail with nodal extensions (22). The head and proximal body of SAN usually are situated subepicardial, near the SVC orifice and beneath the adipose tissues, varying from 10 to 20 mm in length. In contrast, the remaining body and tail portions penetrate obliquely into the musculature of the crista terminalis and are located toward the endocardium (22, 23). The transitional cells regarded as histological and electrophysiological characteristics intermediate between SAN cells and atrial myocytes from the head and body of the SAN extend into the crista terminal crest, the entrance of the inferior vena cava, or occasionally the muscular sleeve of the SVC (24). These anatomical observations were in line with our present findings, in which some cases of SAN below the RA-SVC were from the middle to superior RA. However, the location of the SAN was increased within the SVC in some patients. In addition, Yamashita et al. performed ultra-high resolution mapping of the SVC in a relatively small sample of patients with AF. They showed that 25% (10/40) of patients had a SAN located above the RA-SVC junction (10). Whereas Tanaka et al. reported that none of 113 patients with AF presented the SAN within the SVC using ultra-high resolution mapping (25). However, in our study, in nearly 60.5% of PsAF patients, SAN was located above the RA-SVC junction. The main difference between the above-mentioned studies and the current study is the variation in defining the RA-SVC junction. In Tanaka et al.’s study, the SVC angiogram in all patients was taken with a manual injection of contrast media. The junction of the convex RA wall and the straight SVC wall on the angiogram was defined as the radiological RA-SVC junction. While the definition of the RA-SVC junction in Yamashita et al.’s study was not mentioned. We defined the ostium of the SVC where the diameter of the SVC abruptly changed in the created geometry from cranial to caudal direction as the electroanatomic RA-SVC junction. A second important difference between the results presented by Yamashita et al. and Tanaka et al and our results arise from the selection of patients with AF and the proportion of paroxysmal and non-paroxysmal AF. A third disparity is that RA-SVC construct models based on the different three-dimensional mapping systems. The former two studies used the Ryhthmia system while we employed the CARTO3 system. Finally, the location of the SN can change depending on the autonomic state (26). The average distance between the SAN and the RA-SVC junction was 10.2 mm. In the setting of the SVCI ablation line for these patients, attention should be paid to the SAN position to avoid damaging the SAN, and the ablation line should be as far away from the SAN location as possible. It has been reported that extension from the RA-SVC junction by more than 10 mm may effectively preclude ablation of SAN and perinodal tissues (9). However, our study found that the SAN was located more than 10 mm above the RA-SVC junction in some patients with AF. When performing SVCI, the safe distance between the isolation line and the RA-SVC junction was not enough. Therefore, delineating the location of SAN is helpful to preset a safe ablation pathway.

### Clinical implications

Sinoatrial node injury is the major complication during SVCI that must be avoided. It usually occurs when an RF application is applied too close to the SAN region. Identifying the location of the SAN can help us avoid SAN injury during SVCI. Previous studies have reported that SAN injury occurred during SVCI in 2–4.5% of cases (8, 9). Our study confirmed the feasibility of using the 3D activation mapping system to visualize the detailed SAN location as an adjunctive strategy to SVCI. We found identification of the SAN location in patients who had them very useful because almost 52.9% of SAN locations were situated just above the RA-SVC junction, allowing us to forego unnecessary RF delivery near the SAN. Therefore, the ablation site should be far away from the SAN to avoid the SAN injury. Our results also provide the anatomical basis for the design of the SVCI pathway and improve the safety of SVCI.

## Limitations

There are some limitations to our study. First, we failed to map the EAA of SAN from epicardium because of limitations in the current technology and did not have additional data to further probe the activation sites in patients with sinus dysfunction or heart failure. However, Joung et al.’s studies may have provide an insight into the activation sites in patients with sinus dysfunction. They found that the superior sinoatrial node in patients with AF and symptomatic bradycardia had no response to sympathetic stimulation (27, 28). Further studies are needed to pay attention to this issue. In addition, our study located the farthest distance of SVC muscle sleeve potential and did not further analyze the SVC myocardial sleeve potential in multi-dimensional space. Some studies have demonstrated that SVC myocardial extension was asymmetric (21), which may also be one of the reasons for the heterogeneity of LSVC in various studies. Finally, the study population, especially the patients with PsAF and non-AF, was relatively small. Although no statistical difference in the distribution of sinoatrial node was reached, it is possible that there would be a difference if the sample size was increased.

## Conclusion

The EAA of SAN was located in the SVC in most of the patients, especially in patients with PsAF. The assumed pathway of SVC ablation should be noted at this anatomical location of SAN.

## Data availability statement

The raw data supporting the conclusions of this article will be made available by the authors, without undue reservation.

## Ethics statement

The studies involving human participants were reviewed and approved by The First Affiliated Hospital of Nanjing Medical University, Nanjing, China. The patients/participants provided their written informed consent to participate in this study.

## Author contributions

FZ contributed to the interpretation of data for the work. XC, YL, and YML contributed to drafting the work. QC, HC, WJ, GY, and YZ contributed to data acquisition. PZ, JZ, and YM contributed to the analysis and revision of the work. XS and MC contributed to the conception of the work. All authors contributed to the article and approved the submitted version.
